# Experiences of oral health: before, during and after becoming a regular user of GC Tooth Mousse Plus^®^

**DOI:** 10.1186/s12903-020-01360-8

**Published:** 2021-01-07

**Authors:** Alexandra Sbaraini, Geoffrey G. Adams, Eric C. Reynolds

**Affiliations:** Oral Health Cooperative Research Centre, Melbourne Dental School, Bio21 Institute, The University of Melbourne, Melbourne, VIC 3010 Australia

**Keywords:** Tooth mousse plus, Qualitative research, Grounded theory, Compliance, Adherence, Oral hygiene

## Abstract

**Background:**

Clinical trials and laboratory studies from around the world have shown that GC Tooth Mousse Plus^®^ (TMP) is effective in protecting teeth from tooth decay and erosion, buffering dental plaque pH, remineralising white spot lesions and reducing dentine hypersensitivity. However, no other study has assessed the experiences of oral health, before, during and after individuals becoming regular users of TMP. The aim of this study was to identify how participants’ oral health status changed after introducing TMP into their oral hygiene routine.

**Methods:**

A qualitative study using Charmaz’s grounded theory methodology was conducted. Fifteen purposively sampled regular users of TMP were interviewed. Transcripts were analysed after each interview. Data analysis consisted of transcript coding, detailed memo writing, and data interpretation.

**Results:**

Participants described their experiences of oral health and disease, before, during and after introducing TMP into their daily oral hygiene routine, together with the historical, biological, financial, psychosocial, and habitual dimensions of their experiences. Before becoming a regular user of TMP, participants described themselves as having a damaged mouth with vulnerable teeth, dry mouth, and sensitivity. Various aspects of participants’ histories were relevant, such as, family history and history of oral disease. Having a damaged mouth with vulnerable teeth, dry mouth and sensitivity was explained by those elements. Despite some initial barriers, once being prescribed TMP by a dental professional, a three-fold process of change was initiated: starting a new oral hygiene routine, persevering daily, and experiencing reinforcing outcomes. This process led to a fundamental lifestyle change. Participants transitioned from having a damaged mouth with vulnerable teeth to having a comfortable mouth with strong teeth; at the same time participants felt empowered by this newly found status of being able to keep their teeth for life. Barriers and facilitators for incorporating TMP on daily oral hygiene routine were also identified.

**Conclusions:**

Participants valued having a comfortable mouth with strong teeth, which did not require repeated restorations. Seeing concrete results in their mouths and experiencing a more comfortable mouth boosted adherence to daily applications of TMP, which was maintained over time.

## Background

### The context of this study: adult Australians who were prescribed GC Tooth Mousse Plus^®^ by a dental professional

This study was designed to understand how everyday adult Australians became regular users of GC Tooth Mousse Plus^®^ (TMP). To the best of our knowledge, there is no detailed data on the characteristics of regular users of TMP; hence the context of this study is based on data from oral health population surveys, as described below.

In Australia, around 51% of adults brush their teeth twice a day, with brushing habits declining as people age [1]. Most people pay for their own dental treatments (including home care recommended products) or pay for the private health insurance that partly covers the cost of dental care [2]. Most adults visit a private general dental practice for a check-up at least once a year on average; residents outside capital cities visit less frequently [2–4]. Most individuals visit the same private dental practitioner on a long-term basis [3, 4]. Financial burden is often cited as a reason why people do not visit a dental practice regularly or comply with proposed treatments [4]. Hence, based on population surveys and for the purpose of this study, an average adult Australian would be someone who (a) performs oral hygiene once to twice a day and (b) visits a dental practice at least once a year for a review appointment. In addition, our target population had a particularity: individuals who were prescribed TMP by their dental professional (e.g. a dental professional being a dentist, a dental hygienist, a dental therapist, or an oral health therapist) and became regular users. That is, this study focused on TMP regular users functioning in a typical Australian context in relation to dental treatment and home care.

### What do we know about oral health care compliance and how does it relate to this study?

The term “compliance” is commonly used in dentistry to describe a patient’s readiness and commitment to follow recommendations and instructions [5, 6]. Compliance can be expected in relation to treatment provided in the dental practice or to oral health care instructions to be ensued at home. Wilson described compliance as “the extent to which a person’s behaviour coincides with medical or health advice” [7]. However, it is well known from the dental literature that people frequently “perceive oral health care instructions as difficult to follow and time-consuming” [8]. Therefore, at the beginning of this study we assumed that individuals’ compliance would be key to reach the desired outcome of incorporating TMP into their daily oral hygiene routines. We also assumed that we could identify reasons for non-compliance, such as socio-economic factors, uncertainty about the product, competing priorities and existing habits as previously mentioned in a study looking at compliance to preventive protocols [9].

### What do we know about the product?

The product has been available in the Australian market since 2006. TMP is distributed globally by GC Corporation and GC America. In Japan, Europe, the United States of America, and South America, TMP is known as MI Paste Plus^®^. TMP contains a milk-derived protein called RECALDENT^®^ with incorporated fluoride (CPP-ACPF: Casein Phosphopeptide-Amorphous Calcium Phosphate Fluoride). The level of fluoride in TMP is 900 ppm.

RECALDENT^®^ or Casein Phosphopeptide-Amorphous Calcium Phosphate (CPP-ACP) is a unique ingredient derived from naturally occurring protein found in cow’s milk. RECALDENT^®^ is exported worldwide and used as ingredient in various functional foods and dental products. The CPP-ACP technology is the result of many years of research at the University of Melbourne into the anticariogenic properties of milk. There is a high-level of evidence supporting the ability of CPP-ACP to remineralise early caries lesions and prevent their progression [10–14]. There are now twelve published systematic meta-analyses of these clinical studies to support the use of CPP-ACP to lower caries risk [11–22]. In addition, CPP-ACP effectiveness has also been demonstrated in relation to the reduction of cariogenic bacteria and increased colonization of commensal microorganisms, and the reduction of dentine hypersensitivity [23–26].

**Fig. 1:**
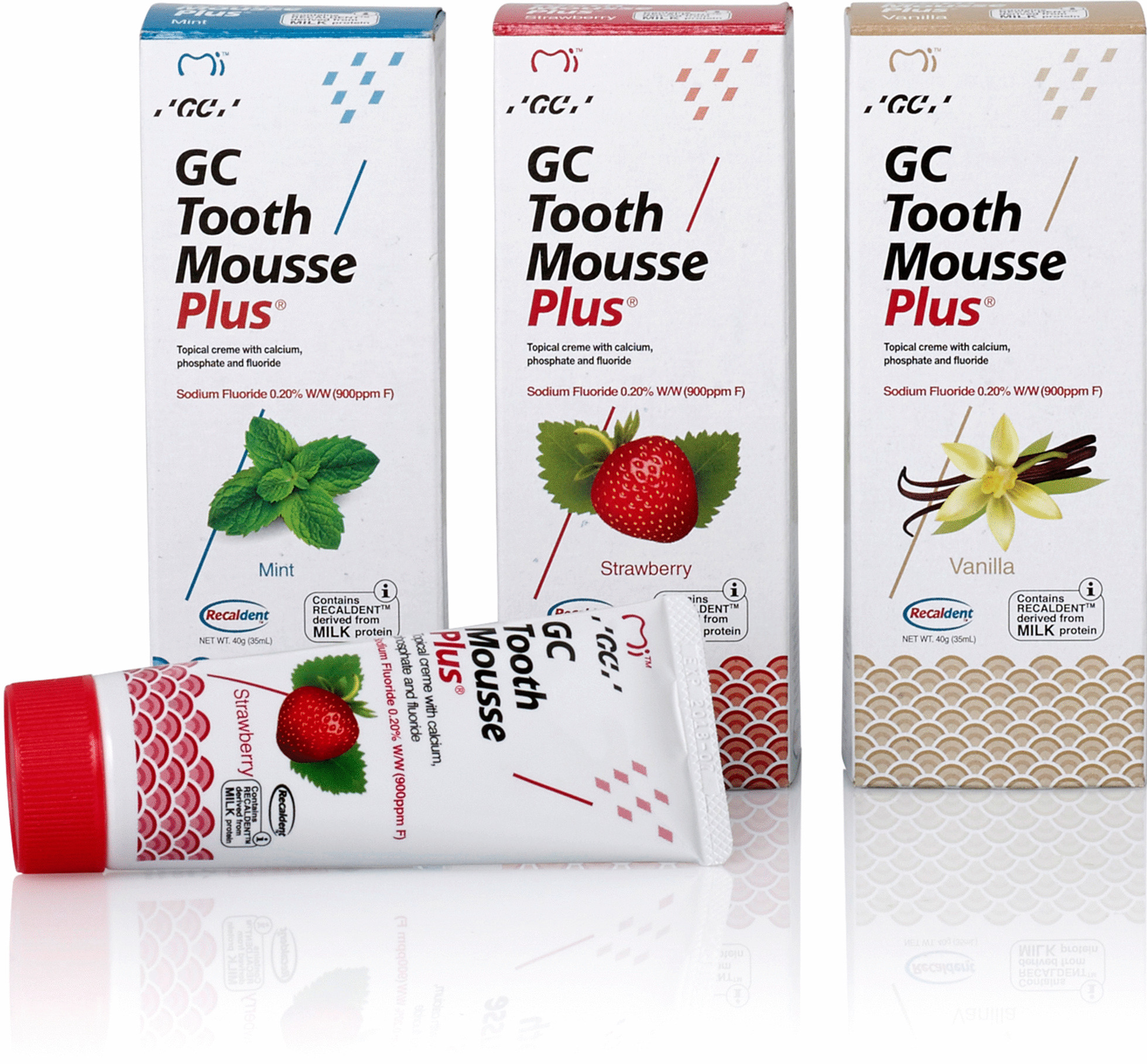
GC Tooth Mousse Plus^®^

In brief, calcium, phosphate, and fluoride within TMP are available in a soluble form—this means the product provides extra protection for teeth, buffers dental plaque acid from bacteria in the mouth and protects teeth from acidic foods and drinks. A dental professional can advise on how often and for how long one should apply TMP. Application should occur after brushing teeth. It is important to acknowledge that TMP does not replace the use of fluoride toothpaste. TMP is available in strawberry, vanilla, and mint flavours (Fig. 1).

### What do we know about consumers’ expectations when applying GC Tooth Mousse Plus^®^?

While the effectiveness of RECALDENT^®^ (CPP-ACP) is well-established in the dental literature, it is not understood how regular users experience incorporating TMP into daily oral hygiene routines. For example, what hurdles they might encounter when asked to change their routines and what is important and valued by them during such process.

In this paper, we report on findings from a qualitative study of TMP regular users’ experiences while adding the product into daily oral hygiene routines, guided by the below research questions:

1. What was participants’ experience of oral health before starting to apply TMP?
2. What was participants’ experience of oral health after starting to apply TMP?
  - How has participants’ oral status changed?
  - How do participants feel about this change?
3. What did participants value in their oral status after starting to apply TMP?

## Methods

### Study design

The design of this study was based on an established systematic methodology: grounded theory procedures [27]. Grounded theory uses a methodically applied set of processes to generate rather than test theory [28]. According to grounded theory procedures, questions are asked in an ‘open’ way: participants’ points of view are sought at commencement rather than questions being asked to examine pre-existing hypothesis or theories [27, 28]. Accordingly, we sought to learn from participants how the process of becoming a regular user of TMP happened and how they made sense of it.

### Sampling strategy

Qualitative studies such as this one typically collect large amounts of data from a small number of participants: there is a trade-off between the number of informants and a wealth of detailed communications with each participant [28]. This study was not designed to estimate proportions in a wider population, quantify relationships between pre-determined variables, or provide a single representative or average view or opinion [28]. Instead, we sought to learn from participants how they experienced the introduction of TMP into daily oral hygiene routines and how they interpreted this experience as explained through their views, needs, values and beliefs.

The target population for this study were consumers who had been regular users of TMP for at least six months prior to recruitment. Being a regular user of TMP meant applying the product daily, at least once a day. An important strength of this study is that we had an agreement with an Australian online supplier of TMP, the BreezeCare Oral Health Company (called BreezeCare from here on). BreezeCare worked in partnership with the research team, so that consumers who bought TMP regularly could be invited to participate in this study. BreezeCare’s database contained individuals from different geographic locations and socio-economic backgrounds, including the most advantaged and disadvantaged areas in Australia. BreezeCare’s database was purposively selected as the company was the major online retailer of TMP. However, it could be argued that a limitation of this sampling strategy was not having access to potential participants who do not purchase TMP from BreezeCare (e.g. individuals who acquire the product from dental practices).

### Sample recruitment

Recruitment was done in partnership with BreezeCare. BreezeCare was responsible for sending the recruitment email. One email was sent to 10,000 customers (these were the total number of customers in the BreezeCare’s database, who had been regular buyers of TMP for at least six months prior to recruitment). The text for the email was prepared by the research team and provided to BreezeCare for writing the email. Table 1 shows text used in the email blast communication. The email blast stated that only the first 50 respondents could take advantage of the incentive to participate because 50 was the number of tubes available from the manufacturer, and there were no research funds available to increase such number.

**Table 1:** Text for email blast communication with regular users

The University of Melbourne in conjunction with GC, the dental company which manufacturers GC Tooth Mousse Plus^®^, would like to know about your experience of using GC Tooth Mousse Plus^®^. In brief, you will be asked why you were prescribed this product, what difference did this product makes to your teeth/your mouth, and for any suggestions you might have for the manufacturer of this product If you are a regular user of GC Tooth Mousse Plus^®^ and are happy to share your opinion, you are invited to be part of a research project called “Experiences of oral health and disease, before and after GC Tooth Mousse Plus^®^”. This research is being conducted by the Melbourne Dental School at The University of Melbourne Participation in this research project is voluntary and you have the right to withdraw at any time, you may also withdraw any data you have supplied. You will not be identified in any publication arising from the research. You will be informed about any publications arising from this research If you are a regular user of GC Tooth Mousse Plus^®^ and are happy to share your opinions by participating in this research, all you will need to do is to send a SMS to Dr Alexandra Sbaraini on [mobile number] * or send an email to [research email] *. Dr Sbaraini will explain the research and collect your feedback on the product Free tube of GC Tooth Mousse Plus^®^ As a "thank you" for your participation, GC will provide you free tube of GC Tooth Mousse Plus^®^. This offer is limited to the first 50 people who contact Dr Sbaraini and it will run for 7 days from [date] to [date]	

^*^Details were omitted for privacy reasons

After the email blast was sent, 35 of BreezeCare’s customers contacted the main researcher (AS), either via text message or email. Then, the main researcher contacted via phone call each of the 35 BreezeCare’s customers to explain the study. In addition, a plain statement description of the study was emailed to the 35 BreezeCare’s customers. The plain statement described what the study was about, what the interview themes were, voluntary participation and withdrawing, project funding, conflict of interest and provided human ethics contact information.

Once the study was described, each customer was asked to reply to AS about their willingness to participate. After the initial communication,16 out of the 35 BreezeCare’s customers did not reply to AS, despite follow-up attempts from the researcher. At the time of recruitment, it was explained to each participant that although they were being recruited then, their respective interview would only occur in a few weeks or months—we anticipate that this may have influenced some participants not to return the researcher follow-up attempts (e.g. emails and phone messages). Nineteen out of the 35 BreezeCare’s customers agreed to participate and provided informed consent. However, four out of the 19 initially recruited dropped out: three participants dropped out because they did not have time availability for an interview and one because her child was the user of TMP, instead of her.

### Sample size and saturation

Sample size in qualitative studies is determined by reaching a complete understanding of the problem being studied—referred to as saturation [27–29]. Saturation is determined by the data analyst. When new interviews become repetitive with prior interviews and central concepts are fully understood, the analyst determines that saturation was reached [29]. In this study, data collection ceased when all the important concepts arising from the analysis were fully understood. Saturation was reached at 12 interviews. Data from the last three interviewees confirmed our findings rather than added new concepts. A total of 15 participants, ranging in age from 25 to 65 years or older, participated in the interview process. Participants characteristics are illustrated on Table 2. They were from four states and one territory in Australia. Participants had different socio-economic backgrounds and different reasons to apply TMP. The majority brushed their teeth twice a day and visited a dental practice twice a year.

**Table 2:** Characteristics of participants (n = 15)

ID	Gender	Age range	Regular user since	Reason for applying TMP	Index of Relative Socio-economic Advantage and Disadvantage (IRSAD)^a^	State and geographic remoteness^b^	Visits a dental practice	Toothbrushing frequency
1	Female	65 years or older	2014	Dry mouth	Quintile 1	NSW, major city	Once a year	Once a day
2	Female	45–54 years old	2016	Dry mouth, erosion	Quintile 3	SA, major city	Once a year	Twice a day
3	Female	45–54 years old	2013	Sensitivity	Quintile 5	NSW, major city	Once a year	Twice a day
4	Female	55–64 years old	2006	Brittle teeth, sensitivity	Quintile 5	NSW, inner regional	Twice a year	Twice a day
5	Male	55–64 years old	2012	Dental caries, sensitivity	Quintile 4	ACT, inner regional	Twice a year	Twice a day
6	Male	45–54 years old	2016	Dry mouth	Quintile 1	VIC, inner regional	Once a year	Twice a day
7	Female	65 years or older	2006	Dry mouth	Quintile 5	QLD, inner regional	Twice a year	Twice a day
8	Female	65 years or older	2010	Dental caries	Quintile 5	VIC, major city	Twice a year	Twice a day
9	Male	25–34 years old	2012	Dental caries	Quintile 4	NSW, major city	Once a year	Twice a day
10	Female	55–64 years old	2016	Dental caries	Quintile 2	NSW, major city	Once a year	Twice a day
11	Male	65 years or older	2015	Sensitivity, dry mouth	Quintile 4	NSW, inner regional	Once a year	Twice a day
12	Female	55–64 years old	2015	Sjogren's syndrome	Quintile 5	NSW, major city	Twice a year	Twice a day
13	Male	35–44 years old	2015	Prevention	Quintile 5	NSW, major city	Twice a year	Twice a day
14	Male	45–54 years old	2012	Dry mouth	Quintile 4	NSW, inner regional	Twice a year	Twice a day
15	Male	45–54 years old	2012	Erosion	Quintile 3	QLD, inner regional	Twice a year	Once a day

^a^IRSAD scale ranges from quintile 1 (most disadvantaged) to quintile 5 (most advantaged)

^b^Australia is divided into five classes of remoteness based on a measure of relative access to services: major city, inner regional, outer regional, remote, and very remote areas

### Interviews

In-depth semi-structured individual telephone interviews were conducted by AS (who has experience in conducting telephone interviews). Telephone interviews are of comparable length, content and quality to face to face interviews, as reported elsewhere in the literature [30–35]. Approximately two to three one-hour interviews were conducted every month over six months with concurrent data analysis. The semi-structured interviews were digitally recorded, professionally transcribed in detail, and the transcripts were checked against the recordings. Table 3 lists questions that guided interviews. The interview questions were developed based on a previous research conducted by AS [9]. Based on the quality and quantity of the information previously obtained, we had a sound understanding about what questions could be helpful in gathering rich data on home care strategies and related experiences.

**Table 3:** Examples of questions asked during interviews

Opening questions	Everyone’s experience of oral health is different. In your own case: When I say oral health, what is the first thing that comes to your mind? How important to you is your oral health and why?
Transitional questions	From now on, we are going to talk about your oral hygiene routines Over the past few years, your dentist or dental hygienist/therapist have prescribed GC Tooth Mousse Plus^®^ as home care therapy. Tell me about your experience of using GC Tooth Mousse Plus^®^ Can you tell me the story of how you found out about GC Tooth Mousse Plus^®^? Why were you prescribed GC Tooth Mousse Plus^®^? So, once you knew about GC Tooth Mousse Plus^®^, what difference did it make for you? What difference did GC Tooth Mousse Plus^®^ make to your teeth/ your mouth? What made this home care therapy easier to follow? What made this home care therapy harder to follow? Do you consider your oral health to be different now vs. prior to using GC Tooth Mousse Plus^®^? How has it changed? What do you think made it change? Who and what was important in this process? How do you feel about this change? What are the most important/relevant tasks that you performed while adopting GC Tooth Mousse Plus^®^ into your daily oral hygiene routine? If we could only make one change in GC Tooth Mousse Plus^®^, what would be the most important change to make? Some people have suggested that all dentists should prescribe GC Tooth Mousse Plus^®^ to all patients. What do you think about that?
Concluding questions	Now I am just going to sum up what I think I have learned about your experiences [SUM UP HERE]. Does that sound right? Is there something else you think I should know to understand your experience of using GC Tooth Mousse Plus^®^? Is there something you would like to ask me?

The interview was divided into opening questions, transitional questions and concluding questions (Table 3). Interviews included open-ended opening questions about oral health (When I say oral health, what is the first thing that comes to your mind? How important to you is your oral health and why?). Then, transitional questions were asked about the experience of oral health before and after using TMP (Tell me about your experience of using GC Tooth Mousse Plus. Why were you prescribed this product? What difference did this product make to your oral health?). Participants were invited to talk specifically about their own experience in applying the product, their perceptions, and expectations about the product efficacy and what changes they have observed in their oral health status after starting to use the product. In brief, the interviewer (AS) explored how participants introduced TMP into daily routines, what were their experiences of oral health before, during and after using TMP, and how this process was influenced by their social context.

Participants were interviewed in places and occasions appropriate to them such as in a private office space during a lunch break or at their home when convenient. Participants confirmed that they were in a quiet and private place before interviews took place. Only AS and a participant at a time were present and listening to the content of the interview. Participants were interviewed only once, but they were contacted after the interview when clarification was required and/or for confirmation of researchers’ understanding. A total of 15 interviews were conducted between August 2017 and January 2018.

### Ethics approval and consent

Ethics approval was obtained from the Human Research Ethics Committee at the University of Melbourne (HREC ID: 1748963). As in any ethical study, we ensured that participation was voluntary, that the participants could withdraw at any time, and that confidentiality was protected. All responses were anonymised before analysis, and we took particular care not to reveal potentially identifying details of geographic locations, dental practices participants visited or clinicians who prescribed TMP to them. Informed consent was obtained prior to interviews. After the interview, participants received a tube of TMP as a thank you for their participation.

### Data analysis

#### Coding and the constant comparative method

Charmaz's iteration of the constant comparative method was used during data analysis [27]. This involved coding of interview transcripts, detailed memo writing and drawing diagrams. The transcripts were analysed as soon as possible after each interview. Coding and interpretation of data was conducted by AS, a trained researcher with PhD and experience of qualitative research and grounded theory methodology [28]. Team meetings were held where AS, GGA and ECR discussed data analysis findings and compared their interpretations.

**Fig. 2:**
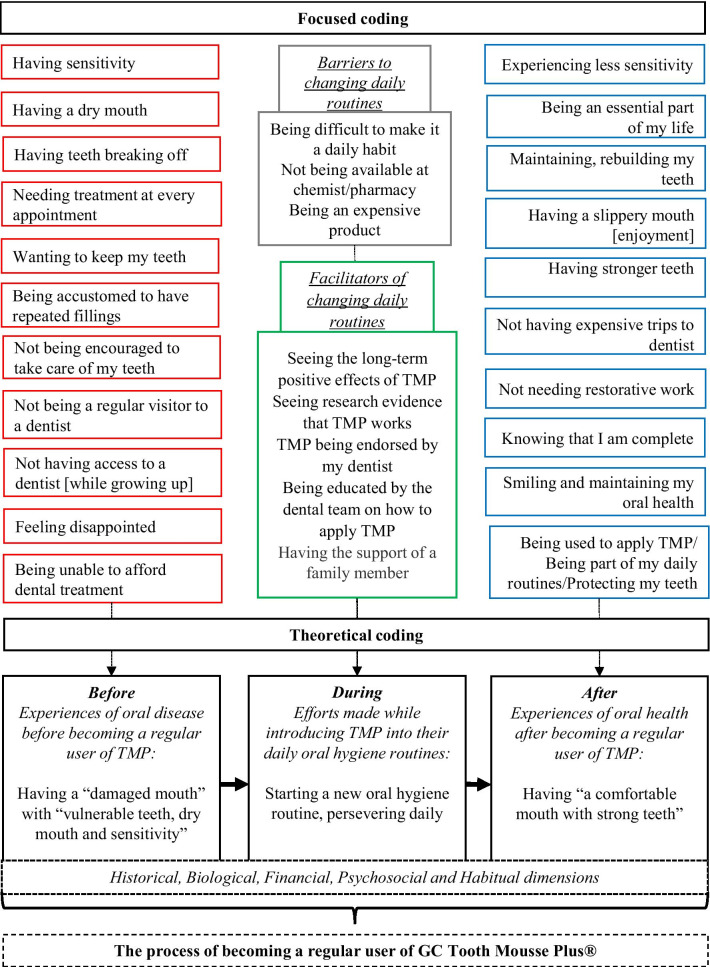
Coding tree

Coding occurred in stages (Table 4). During initial coding, as many ideas as possible were inductively generated from early data. In Charmaz’s form of grounded theory, codes take the form of gerunds (verbs ending in ‘ing’) which emphasises actions and processes [27]. During focused coding, a selected set of central codes was pursued throughout the entire dataset—this process required decisions about which initial codes were most prevalent or important, and which contributed most to the analysis (Fig. 2). During theoretical coding, the final categories were refined and related to one another [27]. As cited by Sbaraini et al., this stage of the coding process entails further analysing each major focused code by examining situations in which such codes emerge, if/when changes occur and how codes relate to one another [28]. Accordingly, data saturation was determined by re-examining the data to gain further insights from several central focused codes. In addition, conceptual memos were written, along with diagrams which allowed researchers to note relationships between codes. We have looked thoroughly for circumstances or events in participants’ narratives which were not explained by the emerging process of becoming a regular user of TMP to expand it further to describe all data. The process of becoming a regular user of TMP presented in this paper is expressed as a set of concepts that are related to one another in an interconnected way and it accounts adequately for all the data we collected (Fig. 2).

**Table 4:** Coding process

Raw data	Initial coding	Focused coding	Theoretical coding
“I want to keep them [teeth] until I take my last breath. I don’t want to have false teeth; I’d rather keep my own teeth …” (ID11, male, 65 years or older, sensitivity,dry mouth)	Wanting to keep my teeth [until I take my last breath] Not wanting false teeth	Wanting to keep my teeth	*Experiences of oral disease before becoming a regular user of TMP*

#### Memo-writing

The primary analyst (AS) wrote extensive memos which documented the development of the codes, what they meant, how they varied, and how they related to the raw data (transcripts). Two types of memos were written: case-based and conceptual memos [27]. Case-based memos were written after each interview—containing the interviewer’s impressions about the participants’ experiences and the interviewer’s reactions—memos were also used systematically to question some of our pre-existing ideas in relation to what had been said in the interview (Table 5). Conceptual memos, on the other hand, were a form of (1) making sense of initial codes; (2) examining participants’ meanings; [3] understanding processes, including when they occurred and changed and what their consequences were. In these memos, data was compared to find similarities, differences and to explain our emerging process (Table 6).

**Table 5:** Case-based memo

Memo written after interview with ID4, female, 55–64 years old, brittle teeth and sensitivity	
This was a very inspirational interview: this lady has such a positive attitude despite all difficulties she faced from childhood to becoming a single mother. She started using TMP because she had brittle and very weak teeth since her childhood. She has also experienced dentine hypersensitivity and had undergone expensive dental treatment in the past. One of her motivations to apply TMP daily is to avoid “big bills” at the dentist Someone who was important during her journey with TMP was a local dentist and co-worker at a country town community health centre—that dentist was the first influencer on her starting using the product. After 6 months of using TMP, she went for a dental check-up and that was the first time in her life she did not need a new restoration done—from that moment she was “sold”. According to her, that day had a massive impact in her life (financially and emotionally): being a single mother, she could no longer afford sparing “a lot of money” for restoring her teeth which were “breaking down” all the time. Consequently, she became TMP greatest advocate in the rural community. She loves this product and encourages her family and friends to use it She truly believes TMP has saved her teeth as they were very brittle and chipped so easily. Her oral health before and after TMP has changed dramatically. She is now confident that her teeth will last through her retirement, if not all but most of them; and for her that means having a renewed confidence in what she can do daily (applying TMP) to take control of her oral health and keep her teeth for life

**Table 6:** Conceptual memo

Experiences of oral disease vs experiences of oral health	
Before being prescribed TMP, participants referred to having experiences of oral disease (e.g. dental caries, sensitivity, painful mouth). While some described having a history of dental caries since childhood, some had a very dry mouth and others had never been offered a different treatment option rather than restorative treatment when visiting a dental practice. So, expecting teeth to break down or requiring a restoration at every dental appointment was the norm. Experiencing oral disease created a certain level of disappointment as participants could only anticipate losing more and more teeth, while their genuine desire was to keep teeth for life When TMP was first recommended to them, despite not being pleased with the position of losing teeth, it was not straightforward for participants to incorporate an additional step into their oral hygiene routine—as it is expected with any change of habit. However, they knew something had to shift if they sincerely wanted to keep their teeth. Feelings such as anxiety, doubt, determination, confidence, and reassurance were part of such process. Consequently, after truly incorporating TMP into their daily routine, participants described distinct outcomes: having experiences of oral health: these included among other things having a more comfortable mouth and considering that they would be able to “keep their teeth for life”(ID4, female, 55–64 years old, brittle teeth and sensitivity) It was clear that participants went through a process of change: without exception, they have described experiencing a profound change in their oral health and in their quality of life because of that. Incorporating TMP into their daily oral hygiene routines was strongly linked to believing in themselves, having support from a dental professional and/or a family member to make the change possible, and believing in the research behind TMP. This process entailed (1) accepting that an additional step was required after tooth brushing and (2) abandoning old beliefs, such as being comfortable with needing repeated fillings and believing this was common (“Isn’t a filling what everyone gets when visits a dentist?”(ID8, female, 65 years or older, dental caries)	

### Reflexivity

Throughout this study, it was important to acknowledge that as researchers we had some pre-existing concepts in mind due to (a) our academic backgrounds in dentistry and (b) our close connection with TMP manufacturer. However, we intentionally stayed open to listen to participants’ narratives. As a result, we were able to engage with participants’ detailed narratives. By intentionally listening to participants’ narratives during data collection and analysis, we were able to delineate the process participants went through with a high level of accuracy.

## Results

During interviews, participants shared their stories at length—they had a clear understanding of what the process of becoming a regular user of TMP entailed and provided a detailed narrative of such process. Participants’ narrative revealed three distinct periods of their lives: before, during and after becoming a regular user of TMP (Table 7).

**Table 7:** The process of becoming a regular user of GC Tooth Mousse Plus^®^

Before *Experiences of oral disease before becoming a regular user of TMP*	During *Efforts made while introducing TMP into their daily oral hygiene routines*	After *Experiences of oral health after becoming a regular user of TMP*
Having a damaged mouth with vulnerable teeth, dry mouth and sensitivity Having sensitivity Having a dry mouth [no saliva] Having teeth breaking off Needing treatment at every appointment Being unable to afford dental treatment Wanting to keep my teeth Feeling disappointed Being accustomed to have repeated fillings Not being encouraged to take care of my teeth Not being a regular visitor to a dentist Not having access to a dentist [while growing up]	Starting a new oral hygiene routine, persevering daily *Barriers to changing daily routines* Being difficult to apply TMP daily TMP not being available at chemist/pharmacy TMP being an expensive product *Facilitators of changing daily routines* Seeing the long-term positive effects of TMP Seeing research evidence TMP being endorsed by my dentist Being educated by the dental team on how to apply TMP Having the support of a family member	Having a comfortable mouth with strong teeth Maintaining, rebuilding my teeth Experiencing less sensitivity Having a slippery mouth [enjoyment] Having stronger teeth Not having expensive trips to dentist Not needing restorative work Knowing that I am complete Smiling and maintaining my oral health Being used to apply TMP/ Being part of my daily routines/Protecting my teeth Being an essential part of my life

Participants described their experiences of oral disease before becoming a regular user of TMP and how they felt about it, physically and emotionally. Before TMP, signs and symptoms of oral disease were very familiar to them. Individuals were living with vulnerable teeth which were brittle, painful, and/or sensitive; some had a dry mouth which led to more discomfort. Participants visited a dental practice once to twice a year; they anticipated a restoration at every appointment and feared paying soaring fees for it (Table 8). It was not a happy life and the simple thought that life could be different did not even cross their minds. On the other hand, after becoming regular users of TMP, they recognized the enormous disparity between life as they knew it (before TMP) and life after becoming a regular user of TMP (Table 9). This change was described by all participants in the study. Historical, biological, financial, psychosocial, and habitual dimensions of their experiences were identified (Table 9).

**Table 8:** Experiences of oral disease before becoming a regular user of GC Tooth Mousse Plus^®^

Having a damaged mouth with vulnerable teeth, dry mouth and sensitivity		
Historical dimension: refers to participants' dental history, their experience of oral disease overtime	Not having access to a dentist while growing up	“Well I grew up in the country, and we were a long way from a dentist; so, I suppose as a small child, I would have seen the dentist once every 10 years.” ID8, female, 65 years or older, dental caries
	Not being encouraged to take care of my teeth	“In my childhood, I wasn't encouraged to take care of my teeth and so I didn't.” ID10, female, 55–64 years old, dental caries
Biological dimension: refers to participants’ experiences of oral health and disease as clinical signs and symptoms	Having sensitivity	"My teeth were quite sensitive; I couldn't eat ice-cream or go out in the cold winter months." ID7, female, 65 years or older, dry mouth
	Having a dry mouth [no saliva]	“I was treated for mouth and throat cancer [radiation therapy], which affected my mouth in a number of ways; it killed off my saliva glands.” ID14, male, 45–54 years old, dry mouth
	Having teeth breaking off	“I couldn’t chew anything without a bit breaking off and I thought that I was looking at getting dentures.” ID3, female, 45–54 years old, sensitivity
Financial dimension: refers to the financial burden of oral disease	Needing treatment at every appointment	“Every time I went to the dentist there was some treatment that needed doing and a cost.” ID7, female, 65 years or older, dry mouth
	Being unable to afford restorative treatment	“I just got so sick of those huge dental bills; I was a single parent…I couldn’t afford to have all the dental work that they were predicting I was going to have.” ID4, female, 55–64 years old, brittle teeth and sensitivity
Psychosocial dimension: refers to the psychological and social aspects of participants’ experiences, including patients’ emotional suffering due to oral disease	Feeling disappointed	“It was disappointing that I just kept cracking my teeth.” ID3, female, 45–54 years old, sensitivity
	Wanting to keep my teeth	“I want to keep them [my teeth] until I take my last breath.” ID11, male, 65 years or older, sensitivity and dry mouth
Habitual dimension: refers to customary activities related to or consequences of oral disease	Not being a regular visitor to a dentist	“I wasn't a regular visitor to the dentist … dental visits were always prolonged, there was more work to be done because I hadn't taken care of my teeth.” ID10, female, 55–64 years old, dental caries
	Being accustomed to have repeated fillings	“I’ve always had to go regularly to the dentist and have surface fillings put on probably nearly all of my teeth over the years. So, I was used to keep getting more and more fillings.” ID12, female, 55–64 years old, Sjogren's syndrome

**Table 9:** Experiences of oral health after becoming a regular user of GC Tooth Mousse Plus^®^

Having a comfortable mouth with strong teeth		
Historical dimension: refers to participants' dental history, their experience of oral disease overtime	Maintaining, rebuilding my teeth	“I believe quite strongly that TMP is helping to maintain my teeth healthy and rebuilding, constantly rebuilding the structure of my teeth to keep them healthy.” ID10, female, 55–64 years old, dental caries
Biological dimension: refers to participants’ experiences of oral health and disease as clinical signs and symptoms	Experiencing less sensitivity	“I don’t really feel a problem of sensitivity at all in my teeth anymore. So, from that point of view, my experience has been that I attribute it to TMP because of the strong correlation between my beginning to use it and the decrease in the problem of sensitivity. So therefore, I just continue to use it, and it has been helpful to me." ID7, female, 65 years or older, dry mouth
	Having a slippery mouth [enjoyment]	“The first thing I noticed orally, it’s kind of a feel good—it makes my mouth slippery; I like that feeling a lot compared to when my mouth is dry and I’m kind of lacking that. So, I use TMP with enjoyment, I look forward to putting it on.” ID14, male, 45–54 years old, dry mouth
	Having stronger teeth	"I kept using TMP because I felt that my teeth were better. Like, the sensitivity was gone, and I felt that my teeth were stronger. I think it is helping the enamel—there aren’t little white spots on my teeth anymore. So, I will keep using it forever.” ID3, female, 45–54 years old, sensitivity
Financial dimension: refers to the financial burden of oral disease	Not having expensive trips to dentist	“The longer I use it [TMP], the less problems I had with things like tooth decay. My dentist hasn’t had to do a filling for me now for over two years.” ID9, male, 25–34 years old, dental caries
	Not needing restorative work	“The first time I went to my dentist for my 6-month check and went out without having to get any [restorative] work I was sold. Do you know what I mean? That was all I needed, I just thought it must be TMP. Then, when it happened again, and then a third time, I just was gobsmacked.” ID4, female, 55–64 years old, brittle teeth and sensitivity
Psychosocial dimension: refers to the psychological and social aspects of participants’ experiences, including patients’ emotional suffering due to oral disease	Knowing that I am complete	“Applying TMP and keeping my own teeth is part of feeling well and knowing that my teeth are part of my body—it's like knowing that I’m complete.” ID11, male, 65 years or older, sensitivity, dry mouth
	Smiling and maintaining my oral health	“I’m excited and I feel like TMP keeps me smiling. I just sense I am doing the right thing in maintaining my oral health.” ID14, male, 45–54 years old, dry mouth
Habitual dimension: refers to customary activities related to or consequences of oral disease	Being used to apply TMP/ Being part of my daily routines/Protecting my teeth	“I think that to keep my own teeth is really important. So, the fact that it takes me a few more minutes to apply TMP is not important to me at all … it’s a bit like exercising every morning before breakfast, so that just becomes part of life. I don’t even think about it now, it’s just part of life. This is tooth protection that I can do at home. It became part of the things that I do every day, like eating meals and so on.” ID11, male, 65 years or older, sensitivity, dry mouth
	Being an essential part of my life	“I think it’s an essential part of my life now—I would never be without TMP.” ID1, female, 65 years or older, dry mouth

### Experiences of oral disease before becoming a regular user of TMP

Before becoming a regular user of TMP, participants described themselves as having a damaged mouth with vulnerable teeth, dry mouth, and sensitivity. Various aspects of participants’ histories were relevant: family history, personal history, and history of oral disease. Having a damaged mouth with vulnerable teeth, dry mouth and sensitivity was explained by historical, biological, and financial elements (Table 8). For example, participants who had grown up without having access to a dentist or who were not encouraged during childhood to take care of their teeth, revealed disappointment about “not doing enough tooth brushing” (ID10, female, 55–64 years old, dental caries) and needing restorative treatment when they eventually visited a dentist. According to them, having a damaged mouth affected their chewing ability as reported by a participant: “I couldn’t chew anything without a bit breaking off” (ID3, female, 45–54years old, sensitivity). Participants believed it was common to have teeth drilled and filled every time they visited a dentist. They were also familiar with the replacement of failed tooth restorations. However, a common finding among their narratives was the fact that they were unable to afford restorative care. This was a major cause of concern and reflected the financial burden participants were facing.

### Barriers to changing daily routines

When TMP was first recommended to participants, despite not being pleased with the possibility of keep losing teeth, incorporating an additional step into their oral hygiene routine was not straightforward (Table 7)—as it is expected with any change of habit. However, they knew something had to shift if they sincerely wanted to keep their teeth. Feelings such as anxiety, uncertainty, determination, confidence, and reassurance were part of such process. Participants spoke about three main barriers: (a) being difficult to apply TMP daily, (b) TMP not being available at chemist/pharmacy and (c) TMP being an expensive product.

The difficulty of starting a new habit was evident for all participants, but among the various reasons one stood out, life itself and its unexpected events including illness, divorce, and death of a loved one:

My TMP use was sporadic for about two months. I would not use it for one week, the next week I would use it once, and in the following week I might use it two or three times. Suddenly, I became a patient [oral and throat cancer] and I have never been sick in my life, so it was like “Why me?” Yeah, so difficult initially to get into the routine, then also my mother passed away, my marriage broke down and I became deeply depressed. It was tough. So, to be honest, to put something extra on the routine was a pain. It became another chore; extra mouth hygiene became annoying. It was hard, and only when the depression started to lift, I saw the importance of looking after my teeth again (ID14, male, 45–54 years old, dry mouth).

Participants also talked about where they purchased TMP (e.g. at dental practices or from online dealers) and how much they paid for it at different locations. Cost was clearly defined as a barrier:

I used TMP very sporadically because I thought it was a bit expensive, and then probably it wasn’t until about five years after that, I started using it regularly (ID4, female, 55–64 years old, brittle teeth and sensitivity).

Obviously, the cost is something that most people, including myself, find it difficult (ID11, male, 65 years or older, sensitivity and dry mouth).

Few participants were happy to purchase TMP from their dental practice; others shifted to online purchase given the price difference between the two settings. While some participants wished they could purchase TMP from a nearby chemist, not being able to do so was also defined as a barrier:

It would be so much easier if you could buy it at a chemist (ID12, female, 55–64 years old, Sjogren's syndrome).

The fact that you could only buy it from the dentist sort of made it a hassle to acquire (ID7, female, 65 years or older, dry mouth).

### The process of becoming a regular user of TMP

Despite encountering initial barriers, once being prescribed TMP by a dental professional, a three-fold process of change was initiated: starting a new oral hygiene routine, persevering daily, and experiencing reinforcing outcomes (Table 7). This process led to a fundamental lifestyle change with five types of outcomes: historical, biological, financial, psychosocial, and habitual. Participants transitioned from having a damaged mouth with vulnerable teeth to having a comfortable mouth with strong teeth. At the same time, participants felt empowered by this newly found status of being able to “keep their teeth for life”. (ID4, female, 55–64 years old, brittle teeth and sensitivity).

### Facilitators for changing daily routines

During this process of change, participants identified key facilitators for changing daily routines to include TMP application. These included: (a) seeing the long-term positive effects of TMP, (b) seeing research evidence, (c) TMP being endorsed by their dentist, (d) being educated by the dentist/dental team on how to apply the product and (e) having the support of a family member.

Participants were drawn to the product because it gave them an option apart from restorative care. Seeing concrete long-term positive effects of TMP in their mouths was a revelation, which positively reinforced daily application of TMP.

Seriously, for the first time in my life, after about two years of using it [TMP], I sort of realised that I hadn’t had a new cavity, a broken tooth, for two years. (ID4, female, 55–64 years old, brittle teeth and sensitivity).

Participants reported that seeing research evidence was important to believe in the product and to start applying it.

The community health dental team were the ones who convinced me that it [TMP] worked. They showed me a lot of research evidence, which showed how well TMP worked and everything, so that was it then; I was sold. (ID4, female, 55–64 years old, brittle teeth and sensitivity).

Equally important was to have dentists and dental team advocating the product and educating participants on how to use it:

My teeth were just on the verge of breaking and he [dentist] said to take this TMP; he explained the chemistry behind it and how it can rebuild your enamel, and how to apply it. I took that tube, I brushed my teeth normally, then I applied TMP every day for almost a year. And then when I went back to the dentist, my enamel was all healed and stronger. So, I swear by the stuff [TMP]. So, even though my teeth are strong now, I go to the oral hygienist once a year and because my teeth are so good, he says he does not need to see me, but I go to the oral hygienist just to get my teeth cleaned, and that is when I buy my TMP. (ID3, female, 45–54 years old, sensitivity)

For one participant, it took a while to become a regular user of TMP but having her husband’s support made it happen.

My husband has encouraged me—he’s taken an interest there. And that is nice, you know. He understands that I need to spend the money and spend the time. (ID10, female, 55–64 years old, dental caries)

For other participant, it was about his parents taking the time to find a different kind of dentist, who would not only fix teeth, but who was dedicated to help him reach a status of having a comfortable mouth with strong teeth. This different kind of dentist took the time, showed, and explained TMP research findings and how the chemistry behind TMP works—these were effective facilitators of change.

When I was younger, I went to the dentist near where I live but, about ten years ago, my parents found a different dentist: he was good. He was kind of different from other dentists; he would not just fix your teeth. He was more about looking at how to prevent deterioration in your mouth. At that time, he said take this product [TMP] to rebuild your enamel (ID9, male, 25–34 years old, dental caries).

### Experiences of oral health after becoming a regular user of TMP

After becoming a regular user of TMP, participants no longer felt that their fate was to have a vulnerable mouth (and all its consequences), as they were able to achieve tangible lifestyle changes. The dimensions shown on Table 8, which had a deteriorating effect in participants’ life, were altered and reinforcing outcomes started to be noticed (Table 9). Participants realised that their dental history had changed: their teeth were stronger, less sensitive and did not require frequent restorations.

When for the first time, during a dental appointment, restorative treatment was not necessary, individuals were astonished by it. They suddenly realised that it was a consequence of becoming a regular user of TMP, as illustrated below:

The longer I use it [TMP], the less problems I had with things like tooth decay. My dentist hasn’t had to do a filling for me now for over two years. (ID9, male, 25–34 years old, dental caries)

The first time I went to my dentist for my 6-month check and went out without having to get any [restorative] work I was sold. Do you know what I mean? That was all I needed, I just thought it must be TMP. Then, when it happened again, and then a third time, I just was gobsmacked. (ID4, female, 55–64 years old, brittle teeth and sensitivity)

It was their accountable daily actions that made it possible. From that moment on, TMP effectiveness was cemented in their consciousness. Hence, experiencing tangible results in their mouths was crucial for truly believing in TMP. Participants described themselves as being responsible for this highly valued status of having a more comfortable mouth. They felt empowered by the sense that they would be able to keep their teeth for life. For example, participants spoke about a newly and invigorating emotional status of feeling complete. Feeling complete or being complete simply meant their body was whole and healthy; a body in which teeth could be maintained. Being complete enabled participants to enjoy life and smile again. Participants stated that applying TMP is “tooth protection” that one can do at home (ID11, male, 65 years or older, sensitivity and dry mouth). Thus, applying TMP became part of their life and it was comparable to daily exercise and eating healthy meals. While reflecting about the reinforcing outcomes noted after becoming a regular user of TMP, participants referred to TMP as being an essential part of their life as reported by a dry mouth sufferer:

“I wouldn’t be without it [TMP] … there’s no other product that I’ve found that is good … I use mouthwash that’s supposed to improve saliva, you can get gels for a dry mouth, none of them are nice to use, so I think it’s just an essential—it’s an essential part of my life now. I would never be without Tooth Mousse” (ID1, female, 65 years or older, dry mouth).

## Discussion

### Transferability of findings and limitations of the study

As with all qualitative research, opinions about the transferability of findings to other locations rely on understanding the context of this study. This was a study of regular users of TMP in Australia. Participants in this study were invited to participate because they were expected to share a wealth of information, rather than being representative of a wider population. All things considered, as in most research, there may be some selection and recall bias resulting from participants having to actively choose to participate in this study, to reply to the invitation email and to remember past events and experiences. In addition, our findings are also limited by the data collection methods we used and our close relationship with the product manufacturer. Below, we address these factors.

Our target sample were regular users of TMP in Australia, who purchased TMP online via BreezeCare website. Although we aimed to recruit participants from all states and territories in Australia (a total of six states and two  territories exist in Australia), participants from only four states and one territory took part in the study. In saying that, we acknowledge that people from the other states and territory might have contributed with different data. Similarly, people who do not buy the product online may respond differently to the questions asked during interviews.

Telephone interviews were our data collection method. In the past, telephone use in social research was not promoted since it was argued that telephone interviews could potentially exclude certain sectors of the population who did not have access to a phone. However, a recent report estimated that only 2% of Australian adults do not to have access to either a landline or a mobile phone, indicating that telephone is a legitimate tool to reach most of the Australian population [36]. In this study, telephone interviews were a valid method of qualitative data collection. Furthermore, potential participants were not recruited through cold calls; prior to the interview, they were contacted by e-mails and messages. Upon voluntary consent, participants engaged in a telephone-based interview. The interviewer, AS, had previous experience with telephone interviewing [28], which was an invaluable resource for this study. Telephone interviews enabled individuals to participate independent on whether they were in major cities or inner regional areas within Australia. It also allowed participants to be interviewed in a familiar environment (e.g. home). In addition, reduced research costs and greater flexibility while booking interviews were observed. On reflection, a project like this could benefit from establishing a toll-free number for call-backs to minimize possible cost barriers for respondents returning calls. Hence, we argue that this study contributes to the growing body of literature supporting telephone interviews as an achievable mode for collecting high quality data [30, 31, 33, 35, 37].

During a qualitative interview, it is crucial to give participants the opportunity to tell their story in their own words. The questions asked should delve into the study aim and be tailored to the participants’ experience [27]. As previously reported, interview’s questions were adapted from a previous study [9] to include this study’s aims and participants’ experiences. Although researchers took care to maintain quality and rigor during such process, one may presume that certain questions could be considered leading to responses. For example, the following question may be considered a leading question…*So, once you knew about GC Tooth Mousse Plus, what difference did it make for you?* Nevertheless, this question was included in our interview script because of the well-established effectiveness of the product in the dental literature. In addition, this question resulted in important data generation which the researchers had not anticipated (e.g. data shown on Tables 6, 7, 8 and 9).

Researchers’ reflections on how we dealt with our close relationship with the product manufacturer were detailed on the Methods section. In this segment, we expand on our reflexivity to address how that relationship might have affected or not the participants’ responses. Participants were fully aware that the study was (1) being supported by the manufacturer of TMP and (2) being facilitated by BreezeCare, the distributor. While this knowledge could have potentially influenced participants’ responses, the researchers could not identify this effect during the interviews. The participants did not report any concerns related to the manufacturer funding the study or the distributor’s assistance via email in recruitment activities. Rather, participants saw it as an opportunity to be listened to: they had important messages they wanted the manufacturer to contemplate, such as product availability at chemists and price reduction. Some participants had new product ideas to share with the manufacturer. Others shared their request for a price reduction from the distributor channel (BreezeCare).

From the recruitment email, participants understood that a tube of TMP would be given to the interviewees after the interview. While there are ethical concerns about offering monetary rewards to research participants, the literature also shows that by offering a non-monetary incentive to participants, researchers are less likely to influence their participation (38). From our experience, offering a tube of TMP did not influence participation, it did not influence participants’ choice of further buying the product (as they were already regular users), but it was a recognition of participants’ time.

### Brief overview of findings and relevance to the dental literature

During this study, we developed a better understanding of how participants experienced disease and oral health. Historical, biological, financial, psychosocial, and habitual dimensions of participants’ experiences were revealed. We saw marked differences between participants’ experiences prior to becoming a regular user of TMP and after having done so. It was clear that participants went through a process of change: without exception, they described experiencing a profound change in their oral health and in their quality of life because of that. Among other things, this process entailed (1) being encouraged to make a change, (2) accepting that an additional step was required (applying TMP daily after tooth brushing) and (3) abandoning old beliefs, such as believing it was common to have teeth drilled and filled every time they visited a dentist.

To the best of our knowledge this was the first attempt at understanding the process of becoming a regular user of TMP. Previous clinical and laboratory studies focused on the effectiveness of TMP in preventing and reversing of early dental caries lesions [10–14]. Meta-analyses of clinical studies have also been published to support the use of the product [11–22]. However, no other study has addressed regular users’ views about the product, how the product performs and addresses their individual needs (e.g. dental caries, dry mouth, sensitivity). More importantly, this study provides new knowledge which might benefit dental professionals when prescribing TMP. For example, this study confirmed that a series of barriers can be encountered when one is asked to start applying TMP. However, despite existing barriers, individuals were able to become regular users of TMP. Overcoming existing barriers was strongly linked to participants having support from a dental professional.

While the focus of this study was not on the relationship between dental professionals and patients, it was evident that the quality of such relationship was essential for participants being encouraged to modify their daily routines. Participants talked about the value of TMP being prescribed by a dentist, and at the same time, being educated by dentists and members of a dental team on how to apply the product. It is well established in the literature that dentists’ and dental team members’ attitude towards patients can impact on treatment acceptance and home care compliance [9, 39–42]. Our findings confirm that having a positive relationship between dental professionals and patients can be a facilitator of change.

Marital status was also important during this process of change: having a supportive partner made it easier to become a regular user of TMP. Accordingly, previous research suggested that marriage supports maintenance of healthy oriented activities [43, 44]. In this study, we considered marital status not only as socioeconomic status, but as a source of social and physical support. Our findings indicate that partners can provide encouragement during the process of becoming a regular user of TMP. Similarly, previous evidence showed that having a partner as a source of support increases motivation for better oral care [45]. However, our findings also demonstrated that participants who were single parents or divorced lacked social support to change oral hygiene routines.

### Compliance versus adherence

At the beginning of this study, we wrongly assumed that individuals’ compliance would be key for becoming a regular user of TMP. Throughout the study, we have learned that becoming a regular user of TMP required a lot more than simply following dental professionals’ recommendations and instructions. Adhering to a new oral hygiene routine meant that participants took an active and independent role in their oral health care. They understood the significance of applying TMP daily. Rather than being obedient and having a passive role, participants took control of their oral hygiene care: they faced and conquered a series of barriers, they made the most of facilitators of change and, at the end, they were able to achieve encouraging outcomes.

## Conclusion

We conclude that, based on the findings of this study, participants seeing concrete results in their mouths and experiencing a more comfortable mouth boosted adherence to daily applications of TMP, which was maintained overtime. Such knowledge provides an important interpretive context for key research-proven benefits of TMP and it can assist dental professionals when recommending TMP to their patients.

## Data Availability

Study data and materials may be made available on request with the appropriate human research ethics committee approval and with the consent of the participants.

## Abbreviations

TMP: GC Tooth Mousse Plus
CPP-ACP: Casein phosphopeptide-amorphous calcium phosphate
CPP-ACPF: Casein phosphopeptide-amorphous calcium phosphate fluoride
